# Oxytocin—a social peptide? Deconstructing the evidence

**DOI:** 10.1098/rstb.2021.0055

**Published:** 2022-08-29

**Authors:** Gareth Leng, Rhodri I. Leng, Mike Ludwig

**Affiliations:** ^1^ Centre for Discovery Brain Sciences, University of Edinburgh, Hugh Robson Building, 15 George Square, Edinburgh EH8 9XD, UK; ^2^ Department of Science, Technology and Innovation Studies, University of Edinburgh, Edinburgh, UK; ^3^ Faculty of Health Sciences, Centre for Neuroendocrinology, University of Pretoria, Pretoria, South Africa

**Keywords:** citation network, oxytocin, social behaviour

## Abstract

In this paper, we analyse the claim that oxytocin is a ‘social neuropeptide’. This claim originated from evidence that oxytocin was instrumental in the initiation of maternal behaviour and it was extended to become the claim that oxytocin has a key role in promoting social interactions between individuals. We begin by considering the structure of the scientific literature on this topic, identifying closely interconnected clusters of papers on particular themes. We then analyse this claim by considering evidence of four types as generated by these clusters: (i) mechanistic studies in animal models, designed to understand the pathways involved in the behavioural effects of centrally administered oxytocin; (ii) evidence from observational studies indicating an association between oxytocin signalling pathways and social behaviour; (iii) evidence from intervention studies, mainly involving intranasal oxytocin administration; and (iv) evidence from translational studies of patients with disorders of social behaviour. We then critically analyse the most highly cited papers in each segment of the evidence; we conclude that, if these represent the best evidence, then the evidence for the claim is weak.

This article is part of the theme issue ‘Interplays between oxytocin and other neuromodulators in shaping complex social behaviours’.

## 1. Introduction

The term ‘social behaviour’ casts a wide net: as generally used, it can encompass any interaction between individuals that contributes to lasting relationships between them. For example, eating might be understood to be a social behaviour if sharing a meal leads to friendship and trust; it is less the behaviour itself that is ‘social’ than the context in which it is performed, and the interpretation placed upon it by the individuals concerned. Thus, social behaviour *can* involve almost any behaviour that has a communicative function, and there are considerable cultural differences in ‘many of the domains that psychologically oriented researchers typically consider, including cooperation, trust, fairness, in-group favouritism/cheating, costly punishment, aggressiveness, morality, and competitiveness’ [1, p. 84].

But those who study social behaviour in animals are generally not speaking of acquired cognitive behaviours, but of behaviours which are apparently ‘pre-programmed’. For example, maternal behaviour in rats is expressed by females after they have given birth and while they are lactating [2]. The mother builds a nest, gathers any young into it, cleans them, crouches over them to allow them to suckle, and will defend the nest and litter against intruders. Such behaviours are species-specific, are not learned, and are expressed only in particular circumstances. A mother rat will gather *any* neglected pups into her nest, responding to distress calls that are specific to the species but apparently not to the individual. By contrast, a ewe will allow only her own lamb to suckle and will rebuff advances from any other.

The expression of maternal behaviour in sheep and rats normally requires the activation of the reproductive tract that accompanies vaginal delivery. This observation suggested the possible involvement of oxytocin, because parturition is accompanied by oxytocin secretion in response to the Ferguson reflex, and because oxytocin is released in the brain as well as into the blood [3]. In 1979, Pedersen & Prange [4] reported that intracerebroventricular (icv) administration of oxytocin to virgin, ovariectomized rats could trigger maternal behaviour if the rats were first primed with oestrogen. Subsequently, Fahrbach *et al.* [5] reproduced this outcome, but others [6,7] did not: it seemed that the effectiveness of oxytocin depended on minor differences in protocols, such as the duration of habituation to the test cage [8]. Moreover, the dose of oxytocin needed (400 ng) was high––more than the total brain content. But then it was reported that, in non-pregnant ewes, icv injections of 5–20 µg oxytocin (albeit also a high dose) could trigger maternal behaviour (acceptance of a lamb), if the ewes had been primed with oestrogen [9], and it became widely accepted that oxytocin was important for maternal behaviour.

Nevertheless, this claim requires qualification. The evidence does suggest that, in rats and sheep, high doses of oxytocin can facilitate the initiation of maternal behaviour, but only in females ‘primed’ by oestrogen and progesterone. By contrast, transgenic mice with no oxytocin [10] or with no oxytocin receptors [11] display apparently normal maternal behaviour, although they are unable to deliver milk to their young.

That oxytocin's effect on maternal behaviour required steroid priming had a precedent: in women, in the hours before the onset of labour, rising oestrogen levels trigger a massive increase in oxytocin receptor expression [12,13], and the sensitivity of the uterus to oxytocin increases about a hundred fold [14].

Thus, for oxytocin to affect ‘social’ behaviours, there might have to be an increase in oxytocin receptor expression in relevant brain areas, as was convincingly demonstrated in the context of sexual receptivity. Oestrogen/progesterone priming in most mammals induces sexual receptivity (lordosis) in females [15,16], and in 1985 it was reported that, in steroid-primed female rats, the lordosis reflex could be facilitated by icv injections of just 1 ng oxytocin [17]. This action was subsequently shown to involve the ventromedial nucleus of the hypothalamus, a site where the expression of oxytocin receptors is controlled by estrogen levels [18], while progesterone interacts directly with oxytocin receptors [19]. But we still need to consider what mechanisms might be involved in triggering a behavioural change.

Oxytocin signalling in the brain is perhaps best thought of as a ‘neurohormonal’ signal [20], whether secreted from dendrites [21,22] or from the varicosities that stud the axons [23]. Magnocellular oxytocin neurons all project to the posterior pituitary, but some also project to diverse forebrain sites including the amygdala [24], where they make synaptic contacts at which they use glutamate as a neurotransmitter. Thus oxytocin neurons speak to different targets in the different languages of both hormones and neurotransmitters [25].

Hormones have *organizational* effects, and/or *activational* effects. In lactating rats, suckling induces dendritic release of oxytocin (an activational effect), which by paracrine actions excites the oxytocin neurons. Importantly, dendritic oxytocin release also has an organizational effect; it ‘primes’ their dendritic stores of vesicles, making them available for activity-dependent release. Priming develops slowly, but once the stores are primed, activity in the oxytocin neurons has a positive feedback effect, leading to synchronous bursts of activity in the oxytocin neurons—and hence to periodic oxytocin release in the hypothalamus that re-primes the dendritic stores, and pulses of oxytocin secretion into the blood to trigger milk let-down [26].

The oxytocin cells response to suckling is, by the explanation outlined above, a consequence of a state-dependent functional re-wiring of neuronal network connectivity. In 2012, Bargmann, reflecting on complex behaviours in invertebrates, wrote: ‘Studies of anatomically characterized circuits in crustaceans and in *Caenorhabditis elegans* suggest that it will not be possible to read a wiring diagram as if it were a set of instructions. Instead, the anatomical connections represent a set of potential connections that are shaped by context and internal states to allow different paths of information flow’ [27, p. 458]. As she noted, neuropeptides may be the instruments of such shaping.

### (a) Oxytocin and affiliative behaviour

That oxytocin could facilitate a ‘social memory’ in sheep suggested that it might be involved in other kinds of affiliative bonds. Prairie voles form exceptionally close bonds; they live in multigenerational family groups: a single breeding pair share a nest and territory, spend a lot of time in close contact, share parental care, and reject intruders of either sex. Their offspring are sexually inactive while part of the family group, but when a young female smells the urine of an unrelated male, she becomes sexually receptive within a day, and after mating forms a long-lasting bond with her partner. The bond can be shown by ‘partner preference tests’ in which a female can choose to spend time with either of two males. By such tests, Insel [28] showed that, in female prairie voles, icv infusions of oxytocin (1.5 ng over 3 h) facilitated the formation of a partner preference even in the absence of mating, while infusions of an antagonist could prevent formation of a mating-induced partner preference. Other vole species such as the montane vole display no mating-induced partner preference; they have a different distribution of oxytocin receptors in the brain.

The conclusion that oxytocin was involved in affiliative bonds prompted the suggestion that it might generally promote social interactions, and in 2005, a paper in *Nature* reported that intranasal oxytocin could promote interpersonal trust in humans [29]. This was to become the highest cited paper ever published on oxytocin [30], and it was followed by several other papers that made similarly bold claims, including that oxytocin can enhance the ability to infer mental states and emotions from facial cues [31], and that it increases generosity [32]. More papers followed in high-impact journals, strengthening the perception that this was a high-impact field, with potential utility in treating conditions such as autism, schizophrenia, anxiety disorders and post-traumatic stress disorder.

The claim that oxytocin is a ‘social neuropeptide’ thus became central to much research on oxytocin. But should we accept it? Research has grown rapidly around this claim, and its diverse strands are assumed to provide solid and independent support. We might review one or other strand of the evidence, but perhaps we need to examine *all* of them to properly evaluate the claim. Here, for each major strand of the evidence, we examine the papers most often cited by other researchers in the field, expecting that these will contain evidence that is thought to be particularly important. If the commonly cited evidence is robust, then perhaps subsequent refinements and extensions have strengthened the claim. But if it is frail, and its frailties have gone unnoticed or been ignored, we must call the whole claim into question.

Here, we mapped the literature relevant to this claim, in a style that we previously used to reconstruct the history of oxytocin research [30]. We searched the Web of Science (WoS) Core Collection for journal articles in English that contain ‘oxytocin’ OR ‘pitocin’ OR ‘syntocinon’ in the title, and searched within that set for articles with ‘social’, ‘prosocial’ or ‘antisocial’ as a topic, meaning that one of these terms appeared in the abstract or among the keywords. We aimed to capture primary evidence, so we excluded items identified by WoS as reviews, and edited the set manually to exclude reviews miscategorized by WoS. This gave 1892 papers published between 1991 and October 2021 that had been cited 88 157 times, including 26 019 times from others of the set. We identified all references between these papers, and used Gephi 0.9.2 to construct a network in which papers are nodes and citations are the links between them [33].

### (b) The beginnings of the network

Early papers on oxytocin and maternal behaviour did not explicitly recognize this as a social behaviour, and the first three papers captured were published in 1991. At that time, as well as being essential for lactation, important in parturition, and involved in electrolyte homeostasis, oxytocin was already well known to be involved in sexual behaviour: it had been reported that, in male rats, icv or peripheral injections of oxytocin could shorten the ejaculation latency [34], and, in female rats, icv oxytocin facilitated the lordosis reflex [17]. These effects involve actions at several sites [35], including, in males, at an ‘ejaculation centre’ in the spinal cord [36], and at peripheral reproductive tissues [37].

One of the 1991 papers, a study of male squirrel monkeys, reported that oxytocin injected icv increased sexual behaviour and aggression, but only in dominant males [38]. Another, reported an association between plasma oxytocin and aggression in patients with disorders of the gastrointestinal tract [39].

The third paper studied social memory. In such experiments, a juvenile rat is introduced into the cage of an adult rat who ‘investigates’ it. The duration of investigation is recorded, and the juvenile removed. Subsequently, the same juvenile is returned into the cage with a new juvenile. Typically the resident will investigate a familiar juvenile only briefly, and the reduction in investigation time is taken as an index of a ‘social memory’ of the juvenile. Popik & Vetulani [40] reported that systemic injections of low doses of oxytocin (approx. 7 ng rat^−1^) impaired this memory.

This surprising outcome seemed consistent with the theory of De Wied that circulating vasopressin acted on the brain to enhance passive avoidance memory while oxytocin had an opposite, amnesic action [41]. De Wied's theory had led to studies on humans using intranasal vasopressin, the first of which showed promise for various conditions of cognitive impairment. However, larger studies failed to replicate these findings [42], and it also became clear that, when vasopressin was given intravenously [43] or intranasally [44], only tiny amounts entered the brain. When it also became clear that peripherally applied vasopressin, by raising blood pressure, produced an arousal that subverted the interpretation of the memory tests, De Wied's theory was left in disarray, and experiments with intranasal vasopressin stuttered to a halt [42].

**Figure 1.**
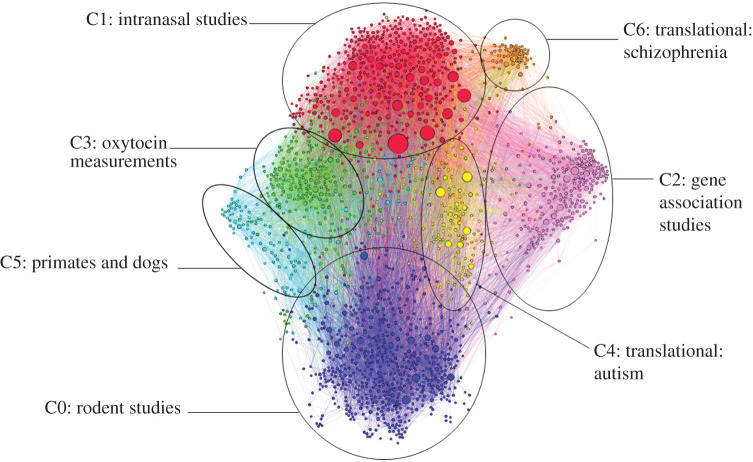
Citation network of articles on oxytocin and social behaviour. The network is composed of 1892 papers (nodes) connected by 26 019 citation links (edges). The nodes are coloured via their cluster membership as determined by modularity maximization (*Q* = 0.43|7 clusters), the circles around each cluster are conjoined to labels that describe the focus of the papers within that cluster. Nodes are sized relative to their indegree, and edges between nodes, represented by lines, are coloured by the colour of the citing paper.

Then in 2005, Kosfeld *et al.* reported that oxytocin, administered intranasally at a dose of 24 IU (approx. 40 µg; a dose close to the total content of the human pituitary), would ‘increase trust in humans' [29]. From this point, interest in oxytocin exploded with the entry of researchers from psychology, psychiatry, neurology and genetics (figure 1).

## 2. Partitioning the citation network

To identify the different strands of evidence from which the claim that oxytocin is a social peptide is constructed, we partitioned the network into clusters by recognizing dense citation links between papers. To do so, we used the Leiden algorithm [45], which recognized seven clusters by the density of citation links between papers. We visualized the network via the ForceAtlas 2 algorithm [46] that clusters densely interconnected nodes together (see [30] for a full description of the methods). In the following account, ‘indegree’ refers to how often a paper appears in the reference lists of other papers in the network; a high indegree thus indicates that a paper has been an influential source of evidence for other primary studies of oxytocin and social behaviour. In the following, we identify papers with high indegrees as an indication of what evidence from each cluster has most influenced other papers in the network.

### (a) Rodent studies

The largest of the seven clusters has 569 papers; these are mostly studies on rats, mice or prairie voles, and are mainly directed at understanding the pathways and mechanisms by which oxytocin affects behaviours. The top six papers, each with an indegree greater than 130, are identified below in bold.

**Insel & Shapiro** [47] reported that oxytocin receptors are distributed differently in the brains of prairie voles and montane voles; among many differences, oxytocin receptors in the lateral amygdala are expressed at high levels in monogamous prairie voles but at low levels in promiscuous montane voles.

**Ferguson *et al.*** [48] used the social memory test to study transgenic mice deficient in oxytocin. These mice display normal sexual and maternal behaviour [10], but this study reported that they are ‘socially amnestic’; they spend as long investigating an unfamiliar juvenile as normal mice, but do not distinguish between familiar and unfamiliar juveniles. Remarkably, this deficit could be corrected by icv injection of 1 ng oxytocin. **Takayanagi *et al.*** [49] reported similar deficits in transgenic mice lacking oxytocin receptors. These two studies marked out the case that oxytocin is important for social behaviour generally, not just for reproductive behaviour.

**Ferguson *et al****.* [50] then showed evidence that the action of oxytocin on social memory is exerted at the medial amygdala. The amygdala is involved in the acquisition, storage and expression of emotional memory, particularly fear memory [51], and **Knobloch *et al***. [52] reported that, in rats, a conditioned fear response could be eliminated by optogenetic induction of oxytocin release in the central amygdala. These two papers thus highlighted the amygdala as a site of particular interest as a key site at which oxytocin affects social behaviour, and suggested that these effects may involve its anxiolytic actions. Accordingly, Churchland and Winkielman, in an influential review [53], questioned whether *all* of oxytocin's effects on social behaviours follow from its anxiolytic actions; they noted that few studies had attempted to control for anxiolytic activity, a remark that still holds true.

The other top paper in this cluster, from **Neumann *et al.*** [54], is widely cited as evidence that oxytocin enters the brain after intranasal administration; this paper is discussed later.

### (b) Intranasal studies

This cluster has 453 papers, mostly studies using intranasal oxytocin in human subjects. Four have an indegree greater than 330, headed by the paper by **Kosfeld *et al.*** on trust [29].

In 2015, a review of studies on trust noted that six studies had failed to replicate the findings of Kosfeld *et al.* concluding that ‘the cumulative evidence does not provide robust convergent evidence that human trust is reliably associated with [oxytocin] (or caused by it)’ [55, p. 772]. Subsequently, the senior author of Kosfeld *et al.* and others registered the protocol for a large, replication study. In 2020, Declerck *et al.* reported its outcome: ‘our preregistered analyses show that the simple and general hypothesis that “[Oxytocin] increases trust” can no longer be endorsed’ [56, p. 651].

In ‘Oxytocin improves “mind-reading” in humans’ [31], **Domes *et al.*** used the ‘Reading the Mind in the Eyes Test’ (RMET); in this test, subjects are shown 36 photographs of faces, and for each they must select which of four adjectives ‘correctly’ describes the emotional state exhibited. Subjects (*n* = 30) were tested after being given 24 IU oxytocin intranasally in one session and vehicle in another; the order was balanced. Subjects scored approximately 27/36 correct responses after oxytocin and approximately 26/36 after vehicle, a difference significant on a one-tailed paired *t*-test. When scores were calculated for only the 18 ‘harder’ test items, the oxytocin ‘effect’ was clearer.

One of the authors co-authored another RMET study, this time with 80 subjects, half of whom had a history of childhood adversity [57]. Again, 24 IU oxytocin and vehicle were given, with an interval of four weeks between sessions. This time there was *no* overall effect of oxytocin in either group, and no subgroup analysis on harder items was reported. However, in the first session (but not the second), subjects given oxytocin performed slightly better than subjects given vehicle. This ‘positive’ finding was highlighted in the abstract.

Other replications of Domes *et al.* have had mixed outcomes [58]; recently, Macchia *et al.* [59] reported that subjects performed *worse* after oxytocin than after placebo. However, authors have been reluctant to suggest that the outcome of Domes *et al.* may be a false positive. Rather, Macchia *et al.* proposed that ‘Given that similar study designs lead to heterogeneous outcomes, our results highlight the complexity of OT effects’ [59, p. 112]. Radke & de Bruijn [60], in their attempt to replicate Domes *et al.,* found that ‘Oxytocin did not affect mind-reading, neither in general nor when considering specific item characteristics’ (p. 75). However, they had assessed trait empathy in the subjects by three measures: none of them correlated with RMET scores before or after oxytocin, but for one there was a significant (*p* = 0.47) negative correlation with ‘RMET performance after oxytocin when controlling for placebo performance’ (p. 78); they highlighted this in their abstract, saying ‘An association between oxytocin-induced changes in RMET performance and emotional empathy … was evident’ (p. 75).

It appears that authors may be shy of declaring that their findings are inconsistent with highly cited papers.

**Kirsch *et al.*** [61], in a neuroimaging study using a region-of-interest (ROI) approach, reported that intranasal oxytocin reduced amygdala responses to images of threatening faces and scenes and reduced its functional connectivity with regions thought to mediate fear behaviour. In subsequent studies, it emerged that oxytocin had opposite effects on the amygdala in men and women, and different effects on different subregions of the amygdala; a review [62, p. 332] noted: ‘despite the initial evidence and hope that oxytocin might reliably decrease anxiety by reducing amygdala activity, subsequent studies have revealed a more complex picture of the modulatory effects of oxytocin on this region’.

In neuroimaging studies, the amygdala seems to be the area most consistently affected by intranasal oxytocin. Generally, this is assumed to reflect a direct action of oxytocin, but inhaled oxytocin reaches the lungs where it is readily absorbed into the blood, and this was once widely used in the management of childbirth at much lower doses than currently used. Because many peripheral tissues contain oxytocin receptors [63], and because intranasal oxytocin raises plasma concentrations to supraphysiological levels, changes in brain activity might be responses to peripheral actions. Recently Martins *et al.* compared the effects of 40 IU intranasal oxytocin on regional blood flow in the whole brain with those of 10 IU oxytocin applied intravenously [64]. Both suppressed amygdala activity, but responses differed at some other brain sites, indicating that not all effects of intranasal oxytocin might be explained by peripheral actions.

**Heinrichs *et al.*** [65] designed a study inspired by the evidence that oxytocin has anxiolytic actions. Healthy men were exposed to psychological stress, involving a mock job interview and mental arithmetic, and salivary cortisol was measured to assess the degree of stress experienced. The men were given 24 IU intranasal oxytocin or vehicle, and half of them were allowed ‘social support’ from a friend. In their abstract, the authors highlight that subjects given both oxytocin and social support ‘exhibited the lowest cortisol concentrations as well as increased calmness and decreased anxiety during stress.’ However, the groups differed in their baseline levels; the lowest increase in cortisol in response to stress was seen not in the subjects given social support and oxytocin, but in those given social support and placebo. This was mirrored in the changes in calmness and anxiety: in subjects given social support, calmness increased more, and anxiety fell more, in subjects given placebo than in those given oxytocin.

Thus, while Heinrichs *et al.* found that intranasal oxytocin was anxiolytic, the evidence for any interaction with social support is questionable. But it is this interpretation, highlighted in the title of the paper (Social support and oxytocin interact to suppress cortisol and subjective responses to psychosocial stress), for which it is mainly cited.

As mentioned, in 2012 Churchland & Winkielman [53] questioned whether *all* of oxytocin's effects on social behaviours follow from its anxiolytic actions, and noted that few studies had attempted to control for anxiolytic activity. One that did is a 2020 study by Kreuder *et al.* [66] which compared the effect of intranasal oxytocin (24 IU) with that of an anxiolytic medication (oral administration of 1 mg lorezepin) on fMRI responses to fear-related images, in a study of young men. Oxytocin and lorazepam both reduced responses in the left central amygdala to fearful faces versus neutral faces, but there were differences in their effects on functional connectivity; in particular, oxytocin produced an increase in functional coupling between the right central amygdala and the left precuneus while processing fearful faces relative to neutral faces. Kreuger *et al.* noted that a similar change had been observed in a study by Eckstein *et al.* [67] during the processing of ‘social stimuli’, and they proposed that, by this change, oxytocin ‘may enhance the salience of social stimuli’. However, it is not clear that this is supported by the evidence cited. Eckstein *et al*. had studied the effect of intranasal oxytocin on a conditioned fear response produced by coupling an electric shock with either a social stimulus (a neutral face) or a non-social stimulus. Intranasal oxytocin facilitated the extinction of the conditioned response—but Eckstein *et al.* state that ‘Auxiliary analysis yielded *no differential effects of oxytocin on social and nonsocial stimuli* during extinction’ (our emphasis) [67, p. 197].

Recently, Mierop *et al.* [68] conducted a systematic review of studies of the effects of intranasal oxytocin on psychosocial outcomes. It reported that tested effects were very heterogeneous; that few replications have been attempted, and most of them were unsuccessful; that significance was unrelated to sample size; and that statistical power was critically low and unrelated to the rate of significant results. Statistical flaws in Kosfeld *et al.* have also attracted detailed criticism in the statistics literature [69]; one issue identified is that the key comparison in Kosfeld *et al*. was, like the key comparison in Domes *et al.*, marginally significant (*p* = 0.025) only on a one-tailed test. Another flaw in Kosfeld *et al.* is one that is common in many neuroscience papers [70]: the effect of oxytocin was judged to be selective for trust because oxytocin had no significant effect on a non-trust test; however, a direct statistical comparison of the effectiveness of oxytocin in the two conditions failed to demonstrate a significant difference [69]. To these statistical weaknesses, we must add a further concern: without predetermined primary outcomes and protocols for data analysis, researchers can analyse and interpret data in ways chosen to promote a particular claim; and the ensuing paper may be cited more for the interpretation advertised by its authors than for the strength of evidence for that interpretation.

### (c) Oxytocin measurements

This cluster has 260 papers, most of which report measures of oxytocin by immunoassays in blood, urine, saliva or cerebrospinal fluid (CSF) in human subjects. Two have an indegree greater than 100.

The top papers were both by **Feldman *et al****.* One measured salivary oxytocin [71], and its abstract states that ‘mothers who provided high levels of affectionate contact showed an [oxytocin] increase following mother–infant interaction but such increase was not observed among mothers displaying low levels of affectionate contact’ (p. 1133). However, overall there was no significant effect of contact, and no statistical test was reported of the difference in responses between mothers with high and low levels of affectionate contact.

The other paper [72] reported a correlation between plasma oxytocin in pregnant women and their subsequent maternal behaviour. It found oxytocin levels of approximately 300 pg ml^−1^—approximately 100 fold higher than generally measured in humans. Most previous studies had measured oxytocin by radioimmunoassay after extracting plasma to remove large proteins that could interfere with the antibody, but these authors used a commercial ELISA without extracting the samples, not noticing that this was recommended by the assay manufacturers. The same problem affects many papers in this cluster. Of the 20 top papers, 10 measured oxytocin in unextracted plasma, reporting mean levels of 198–475 pg ml^−1^; five measured it in extracted plasma, reporting mean levels of 1.6–11 pg ml^−1^. The cluster includes one of the first papers to explore this striking discrepancy [73], finding that levels measured in extracted plasma were uncorrelated with levels measured by the same assay without extraction.

We might reflect on why anyone ever bothered to extract plasma samples since this is expensive and time-consuming. Immunoassays involve incubating a sample with labelled oxytocin and an antibody, and measuring how much of the labelled oxytocin ends up bound to the antibody. Because the labelled oxytocin competes for binding with any oxytocin in the sample, the more oxytocin there is in the sample, the less labelled oxytocin will be bound. However, plasma contains abundant large proteins, including millimolar concentrations of immunoglobulins. Some bind to the oxytocin antibody and, if this impairs its ability to bind oxytocin, the assay will falsely report high levels [74]. This problem of ‘plasma matrix interference’ can be avoided by extracting plasma to eliminate molecules of high molecular weight. Oxytocin levels in extracted plasma samples agree well with bioassay measurements, indicating that they measure biologically active oxytocin [74]. Some immunoassays can accurately measure oxytocin in unextracted mouse plasma [75], but whether *any* can accurately measure it in human plasma is unclear; laboratory mice are inbred and are kept in specific-pathogen-free housing, so have a lower and less variable complement of endogenous antibodies than human populations.

Measurements in extracted and unextracted samples of human plasma give values that differ by at least two orders of magnitude, and which are in many assays uncorrelated [74,76,77]. We may be confident that measurements of oxytocin in extracted plasma report biologically active oxytocin, but what is measured in unextracted plasma is unclear. Some assays might be measuring ‘bound oxytocin’ [78]. Oxytocin does bind to large proteins in plasma, but very slowly (when oxytocin is added to rat plasma and incubated at 37°C, less than 10% of its biological activity is lost within 40 min [79]; when it is added to human serum and maintained at room temperature for 24 h, only approximately 15% is lost [80]). If such binding is irreversible and if bound oxytocin evades the enzymatic degradation that occurs in the passage of blood through the liver and kidneys, bound oxytocin might accumulate to high levels. But why it might be interesting to measure bound oxytocin is unclear, given that its pharmacodynamics and the identity of the binding proteins are unknown.

Confusion was added by a study from Brandtzaeg *et al.* [81] using nano liquid chromatography-mass spectrometry (LC-MS). They processed plasma with a reduction/alkylation step to liberate bound oxytocin, reporting levels of ‘total oxytocin’ (OT-R/A) of 500–1200 pg ml^−1^. But Franke *et al.* [80], using a high-resolution accurate-mass (HRAM) orbitrap-based LC-MS method, cast doubt on this procedure. Using the same reduction/alkylation step, they also (at first) found very high levels of OT-R/A in human serum, but with low precision. After narrowing the mass window, they were unable to find any OT-R/A but found large signals for masses greater than or equal to 8 ppm different from OT-R/A; they concluded that measurements using a reduction/alkylation step were confounded by this interference.

### (d) Gene associations

This cluster contains 205 papers, mostly studies of associations between oxytocin receptor genes and psychological characteristics. Four have an indegree greater than 100.

The top paper, by **Rodrigues *et al****.* [82], is one of several reporting on rs53576, a single-nucleotide polymorphism (SNP) of an adenine (A) or guanine (G) in the oxytocin receptor gene. Other top papers had reported that individuals with one or two copies of the A allele were more likely to be diagnosed with autism [83], and display less ‘parental sensitivity’ [84] than individuals homozygous for the G allele. Rodrigues *et al.* reported that individuals homozygous for the G allele scored higher than all others on the RMET, on a self-report measure of dispositional empathy, and on two measures of stress reactivity. However, a subsequent meta-analysis of studies with a combined sample size of greater than 8000 found no effect of rs53576 genotype on generalized empathy, cognitive empathy or affective empathy [85], and a recent preregistered study found no association between variation in rs53576 and Theory of Mind ability [86].

In 2007, a Working Group on Replication in Association Studies noted that ‘comprehensive reviews of work based on the candidate-gene approach, have demonstrated a plethora of questionable genotype–phenotype associations, replication of which has often failed in independent studies’ [87, p. 655].

Several recent papers in this cluster have extended gene association studies to embrace expression of what is claimed to be an ‘oxytocin pathway gene’, CD38 [88,89]. But CD38 is not specific to the oxytocin system; this transmembrane enzyme is expressed in neurons, astrocytes and microglia in virtually all areas of the brain [90], and it has many different roles. One of its roles is in the production of the second messengers adenosine diphosphate-ribose (ADPR) and cyclic ADPR (cADPR). cADPR is a calcium mobilizer, and it is by this action that CD38 affects oxytocin release in the brain [91], as exocytosis of oxytocin-containing vesicles from the soma and dendrites of oxytocin neurons is regulated by mobilization of intracellular calcium stores [22]. By this understanding, it would be expected that CD38 expression *in oxytocin neurons* is indeed an important element in oxytocin signalling pathways. However, the release of vasopressin, dopamine, serotonin and noradrenaline are similarly affected by CD38 [90], and, by its other roles, elevated CD38 expression throughout the brain is linked to ageing, neuroinflammation and neurodegeneration [90].

### (e) Translational studies: autism

This cluster contains 144 papers, mostly studies of patients with autism or related conditions. Five have an indegree greater than 125, and four of these used intranasal oxytocin. The exception, a 1998 paper by **Modahl *et al.*** [92], used radioimmunoassay to measure oxytocin in extracted plasma; it found that a sample of 29 children with autism disorder had lower oxytocin levels than controls. This paper was cited modestly before 2004, but by 2021 had accumulated 499 citations.

Between 2005 and 2020 many other studies measured oxytocin in subjects with autism or related disorders; a 2021 meta-analysis concluded that these indicated a difference in children but not adults [93]. However, this meta-analysis combined data from studies that reported mean plasma levels that differed between 0.6 and 2290 pg ml^−1^. Fourteen studies that assayed unextracted plasma all reported mean levels greater than 100 pg ml^−1^. By contrast, eight studies of children (including Modahl *et al.*) measured oxytocin in extracted plasma and reported mean levels <25 pg ml^−1^; in four of these, the mean was lower in autistic subjects than in controls, and in four it was higher.

The top paper, by **Guastella *et al.*** [94], used the RMET to study effects of intranasal oxytocin (24 IU) in 15 young males diagnosed with Autism Disorder or Asperger's Disorder. Nine scored higher after oxytocin than after vehicle, and the improvement (from approx. 16/36 to approx. 17/36) was statistically significant. **Andari *et al.*** [95] studied 13 subjects, reporting that intranasal oxytocin produced ‘more appropriate social behaviour and affect’, a core dimension of autism.

The next two papers by **Hollander *et al.*** [96,97] both reported on a study of 15 patients, six of whom had been diagnosed with autism and none with Asperger's. In one session, the patients were given intravenous oxytocin (9 IU over 4 h), and in another session vehicle, in a randomized order. One of these papers reported that repetitive activity fell more after oxytocin than after placebo [96]. The other paper measured the ability to assign affective significance to speech [97]. In the first session, patients performed similarly whether given placebo or oxytocin, and better at the end of the session than at baseline. In the second session, patients again performed similarly at the end, but differed at baseline—those given saline in the first session, around 2 weeks earlier, had lower baseline measures than those previously given oxytocin. The authors interpreted this as implying that patients given oxytocin in the first session retained the benefits of the improvement whereas those given saline had not retained the benefits of a similar improvement. No such ‘carryover’ had been seen for repetitive behaviours.

Most studies in this cluster are of small samples with diverse diagnoses, have multiple endpoints, and display flexibility in analysis. However, the cluster includes a large preregistered trial of 106 patients [98] conducted as a replication of a small study that had reported a positive effect of repeated intranasal oxytocin on core symptoms of autism. It found no effect of oxytocin on the core symptoms of autism (the primary outcomes).

In 2021, Sikich *et al.* [99] reported on an even larger preregistered trial of intranasal oxytocin in 250 children and adolescents with autism spectrum disorder. It showed no benefits of daily oxytocin treatment for 24 weeks on the primary outcomes (measures of social or cognitive functioning) or on any secondary outcome.

### (f) Translational studies: schizophrenia

This cluster has 80 papers that are mostly studies of patients with schizophrenia. The top paper [100] (with 227 citations and an indegree of 85) reported on a study of 20 patients, 11 given intranasal oxytocin for 14 days and nine given placebo. The abstract states that psychotic symptoms declined significantly and several social cognition measures improved ‘significantly or nearly significantly’ in oxytocin but not placebo recipients. However, for only one of 13 outcome measures was there a significant difference (*p* = 0.047) between the oxytocin group and the placebo group, raising concerns about the adequacy of correcting for multiple comparisons.

A recent meta-analysis concluded that repeated administration of intranasal oxytocin had no effect on most core symptoms of schizophrenia, beyond a ‘small tentative effect’ on general symptoms [101]. However, looking at the heterogeneity in the responses to intranasal oxytocin, the authors suggested that there might be a subgroup of responsive patients.

### (g) Primates and dogs

The final cluster contains 125 papers that are mainly studies of non-human primates or of dogs; the dog papers often cite primate papers, but the converse is less true.

The papers on dogs mainly concern interactions between dogs and their owners. The top paper [102], with an indegree of 47, involved giving 40 IU oxytocin intranasally to dogs of diverse breeds; this ‘significantly increased the duration that the dog gazed at the owner in female dogs but not male dogs’ (p. 335). Urinary oxytocin concentration increased in the owners of female dogs that received oxytocin even though oxytocin was not administered to the owners.

The papers on primates are mainly studies on cognitive functions that are not feasible either in rodents or in humans. The top paper [103], with an indegree of 79, was a study on rhesus macaques using intranasal administration of 25 IU oxytocin (about 10 times the likely oxytocin content of the pituitary). In repeated tests, monkeys were given the choices of (i) rewarding themselves or another monkey; (ii) themselves or no monkey; and (iii) another monkey or no monkey. In the first 2 h after oxytocin, the monkeys made more ‘selfish choices’, choosing to reward themselves rather than another monkey. But in the next 2 h the monkeys made more ‘prosocial choices’—rewarding another monkey when the alternative was to reward no one. The authors concluded that ‘inhaled OT causally promotes social donation behaviour in rhesus monkeys, as it does in more egalitarian and monogamous ones, like prairie voles and humans, when there is no perceived cost to self’ [103, p. 959]. We have no idea what studies on prairie voles were referred to. The most relevant human study cited by the authors is a highly cited paper on oxytocin and generosity [32], a critique of which [104] revealed, in the words of Churchland & Winkielman [53, p. 395], that it contained ‘dramatic data interpretations and some unorthodox uses of statistics’.

## 3. Reflections

Each cluster appears to host a community with a particular approach to evidence. The rodent studies are *mechanistic*, concerned with establishing how and where oxytocin acts. Intranasal studies are *intervention* studies, they accept that endogenous oxytocin has a role in regulating social behaviour in mammals, and are concerned with establishing exactly what exogenous oxytocin does in humans. These have widespread methodological shortcomings and are beset with uncertainties over how much oxytocin reaches the brain. The oxytocin measurement studies and the gene studies are seeking *associational* evidence, a weak surrogate for causality. They accept that there is good evidence of mechanism and of the efficacy of interventions, and seek evidence that individual differences in social behaviour are associated with differences in sensitivity to oxytocin or in secretion of oxytocin. The measurement studies are plagued by analytical problems and the questionable relevance of peripheral measurements to central activity, and the gene association studies are typically underpowered. The *translational* clusters accept the evidence from intervention studies and are primarily concerned with the potential of oxytocin for treatments, but have produced little robust evidence of any efficacy.

In identifying top-cited papers, we are not implying that any are of high methodological quality or robust evidential value. Several have continued to be highly cited despite having failed replication. This should not be surprising. In many fields of science, common statistical weaknesses and pervasive biases mean that early studies on a new topic tend to be small and unreliable, but are likely to be highly cited. Subsequent larger and methodologically refined studies typically find smaller effect sizes or are unable to replicate the initial findings, but tend to be much less well cited. Replication studies that fail to replicate the initial outcomes are less likely to be published (the ‘file-drawer effect’), and if published are less likely to be cited than confirmatory studies.

While it should be obvious that citation counts do not measure study quality, it is often assumed that ‘the best and most valuable papers will attract more attention, shaping the research trajectory of the field’ [105]. However, citations to highly cited papers are amplified by citations from papers outside the immediate field, from authors who may uncritically assume their reliability. We sought to minimize this distortion by looking only at indegree—citations from other primary papers on oxytocin and social behaviour, from authors that might be expected to be aware of the quality of directly relevant papers. In fact, we found that indegree correlates closely with total citation counts, suggesting that it is not just authors outside the field who uncritically assume the reliability of top-cited papers.

If, in each cluster, the top-cited papers represent its greatest canonical achievements, there would seem to be disappointingly little progress to celebrate. In the intranasal cluster, the six papers with most citations from ‘oxytocin/social’ papers in 2012 were all among the seven top-cited papers in each of the next 8 years. Indeed the twenty papers in this cluster with most citations in 2020 were all published before the end of 2013. It seems that top-cited papers are generally the first to be noticed; but as mentioned previously, first studies are usually small, often unreliable, and even when true generally have exaggerated effect sizes.

Papers in each cluster mainly cite other papers in the same cluster (see electronic supplementary material, table 1), and citations between clusters mostly involve just a few highly cited papers, so awareness of problems in one cluster may not readily penetrate another, as perhaps exemplified by the issues of plasma matrix interference in immunoassays. Some review articles bridge between clusters—but reviews seldom linger on problems but rather summarize what are perceived to be achievements from the perspective of the review author (see e.g. [106,107]).

## 4. Does intranasal oxytocin reach the brain in effective amounts?

The research community is divided between those who think that effects of intranasal oxytocin are more likely to be a consequence of peripheral actions than of oxytocin entry to the brain, and those who believe that the best explanation of the effects of intranasal oxytocin is that, however little oxytocin enters the brain, it must be enough.

One of the top rodent studies (Neumann *et al.* [54]) is widely cited as evidence that intranasal oxytocin enters the brain. It reported that, in rats, intranasal administration of 20 µg oxytocin (a dose approx. 20 times greater than the pituitary content) produced a marked increase in the plasma oxytocin concentration, but *no* significant increase in the CSF (60 ± 13 pg ml^−1^ in oxytocin-treated rats versus 56 ± 17 pg ml^−1^ in controls). In microdialysis samples from some brain regions, extracellular concentrations increased to about twice the basal level—but a microdialysis probe cannot be introduced into a brain area without damaging blood vessels in that area, so the blood–brain barrier is unlikely to be intact at those sites. The same reservation applies to a later microdialysis study in oxytocin knockout mice [108].

The top primate paper reported that, after giving 25 IU oxytocin intranasally to two rhesus macaques, the CSF concentration in each was approximately 50 pg ml^−1^ whereas levels in four monkeys given saline were less than 30 pg ml^−1^. Lee *et al*. [109] tested whether this reflected entry of exogenous oxytocin or release of endogenous oxytocin. They gave 80 IU of labelled oxytocin to rhesus macaques, and measured it in CSF and plasma. Peripheral administration of oxytocin did not lead to central release of endogenous oxytocin, and intranasal administration was no more effective than intravenous administration in delivering oxytocin to the brain. By either route, penetration was modest despite the very high dose used, and was very variable between animals. However, intranasal administration consistently led to a progressive increase in plasma oxytocin to supraphysiological levels (>500 pg ml^−1^).

Clearly intranasal and intravenous routes are not equivalent; while most of the oxytocin delivered intranasally enters the gut, high concentrations enter extravascular compartments. Oxytocin in the blood is rapidly degraded as it passes through the liver and kidneys, but oxytocin in extravascular fluid is relatively stable; it enters the blood slowly, generating a prolonged increase in plasma concentrations.

In another study, Lee *et al.* [110] again gave labelled oxytocin intranasally to macaques, but this time found no labelled oxytocin in the CSF at any timepoint studied. However, they also analysed microdissected brain regions, and in two monkeys given 80 IU they detected labelled oxytocin in the brainstems of both (but in the amygdala of neither), and proposed that oxytocin might have reached the brainstem along the trigeminal nerve.

Overall, Lee *et al.* showed that intranasal administration of oxytocin leads to some entry into the brain, that the degree of entry is very variable, and that the total amount that enters is generally consistent with the estimate of Mens *et al.* [43] that just 0.002% of the applied dose reaches the CSF. The issue is not whether *any* oxytocin reaches the brain after intranasal administration, but whether enough enters to reach effective concentrations at relevant sites. The paper from Mens *et al.* came from the group of de Wied that was committed to the belief that oxytocin and vasopressin readily entered the brain, and the abstract ends with the extraordinary statement ‘*The present data demonstrate that neurohypophyseal hormones do cross the blood-brain barrier in amounts obviously sufficient to induce central actions*’ [43, p. 143]. For some, now as then, it seems that evidence that any oxytocin enters the brain is evidence enough that enough enters to act there.

Despite the many studies on intranasal oxytocin, it remains doubtful whether enough oxytocin enters the brain to account for the behavioural effects. To date, only one study has reported the effects of intranasal oxytocin on CSF levels in humans [111]. It found no elevation at 45 or 60 min after 24 IU, but in three subjects measured at 75 min, CSF levels were 64% higher than the average of placebo-treated subjects.

In 2019, evidence was published from an *in vitro* model of the blood–brain barrier that RAGE (receptor for advanced glycation end-products), expressed in vascular endothelial cells, might transport oxytocin from the blood into the brain [112]. This proposal has yet to be independently confirmed, and appears inconsistent with previous evidence for a saturable oxytocin transport system from the brain into the blood, contributing to the clearance of oxytocin from CSF [113].

The intranasal route has other limitations beyond its limited brain penetrance, the most obvious of which is its inconsistency. Fifty years ago, intranasal oxytocin was commonly used in maternity hospitals to induce or augment labour at concentrations much lower than those used in psychological studies [114]. However intranasal oxytocin has long been abandoned in maternity hospitals because of the erratic delivery compared to intravenous infusion.

Given the variability in how much oxytocin is delivered to the brain, it may seem surprising that so many studies with intranasal oxytocin have had positive outcomes. But across many fields in science, the literature reports many more statistically significant findings than would be expected from the power of the studies concerned. Explanations include publication bias—the reluctance of journals to publish studies with negative findings, combined with the ‘file-drawer effect’ [115,116]—the reluctance to publish studies that seem unlikely to be cited [117]. Another explanation combines confirmation bias with researchers' ‘degrees of freedom’, allowing flexibility in selecting evidence and modes of analysis to derive outcomes consistent with their expectations. When Walum *et al.* argued [118, p. 251] that ‘there is a high probability that most of the published intranasal oxytocin findings do not represent true effects’, they were pointing to weaknesses common to many fields of science [119].

Oxytocin has diverse effects on many peripheral tissues, so how sure can we be that any behavioural effects of exogenous oxytocin are not secondary to one of these? And, as oxytocin affects many different functions and behaviours, how can we be sure that what we are observing is a primary effect rather than an epiphenomenon? We may design experiments to detect changes in trust, for example, but perhaps the outcomes are confounded by effects of oxytocin on appetite, libido, attention, stress or mood.

The case for oxytocin as a social peptide is often presented as the product of evidence from diverse sources. From the present analysis, if the most highly cited papers in most segments of the evidence represent the best evidence, then the case is weak. To critical eyes, the case for this claim will not be strengthened by more studies designed to confirm it. It seems time to design experiments that attempt to refute it.

## 5. Recent papers

In this review, we set out to examine the papers most often cited by other researchers in the field, expecting that these will contain evidence that is thought to be particularly important. We found the evidence in those papers to be frail, but perhaps the papers were cited not because they were thought to be good, but simply to acknowledge the foundations of the field. It seems to be generally true that the first papers in a field are often underpowered, often poorly replicable, and typically report exaggerated estimates of effect size, but are nevertheless likely to be highly cited if the field takes off. As a field matures, subsequent studies may be larger, more sophisticated and better controlled—but are typically much less well cited. The reasons for this are mundane; in the early years of a field, papers will cite a high proportion of the papers in a small source literature. As the field grows, authors have an ever-increasing literature from which to select their references, but still tend to include the already highly cited papers simply because they are highly cited. New papers, however good, will struggle for attention [117]. So, in our final analysis we focused on the 1037 oxytocin/social articles published since the beginning of 2016 as recorded by WoS by October 2021. Their reference lists contained 31 273 documents, and we recorded how often each of these documents was cited by the recent social/oxytocin articles in each year from 2016 to 2021. Of the 31 273 documents, 24 338 were cited only once. Just 367 had an indegree of more than 20, and of these, only 44 were published since 2016.

The four top cited documents published since 2016 were all reviews from the 2016 special issue of *Biological Psychiatry*. Heading the list, with an indegree of 176 is ‘The social salience hypothesis of oxytocin’ by Shamay-Tsoori & Abu-Akel [120]. This review assumes that the reported effects of intranasal oxytocin are all true effects, and summarizes their diversity and apparent inconsistency as effects that ‘appear to be context dependent, and can be both positive and negative’ (p. 200). The authors note that there are different ‘theoretical formulations’ to explain them: that oxytocin mainly enhances affiliative prosocial behaviours; that it affects social performance by attenuating stress; and that it regulates cooperation and conflict among humans in the context of intergroup relations. To reconcile these accounts, they propose ‘a theoretical framework that focuses on the overarching role of oxytocin in regulating the salience of social cues through its interaction with the dopaminergic system’ (p. 194), while noting that how it does so depends on individual differences such as gender and personality traits.

But more critical voices are also prominent in the highly cited papers. Second in the list is ‘Intranasal oxytocin: myths and delusions’ by Leng & Ludwig [63] with an indegree of 130. In third place, Neumann and Slattery [121] covers the case (mainly from rodent studies) for a role of oxytocin in anxiety and fear; it discusses (sceptically) the prospects of using oxytocin as a therapy for social anxiety, noting that chronic oxytocin treatment is likely to produce receptor desensitization and thereby an increase in anxiety. Fourth is Walum *et al.* [118], which argued that there is a high probability that most reported effects of intranasal oxytocin are false positives. The issues raised in these and other critical reviews were not ignored, and some of the key proponents of intranasal oxytocin echoed and amplified some of the messages—in particular the need for awareness of common statistical pitfalls, the importance of preregistering studies intended to be definitive and powering them adequately; and the importance of transparency in reporting and of replication studies [122]. Also now generally understood is the very low degree of penetration of oxytocin into the brain, while peripheral levels are raised supraphysiologically; this concern does not eliminate the possibility that intranasal oxytocin acts in the brain, but emphasizes the need for peripheral actions to be controlled for [64,123]. Finally, awareness of the unreliability of certain assays for oxytocin is now widespread, and there have been new efforts to establish reliable assays, as we have also detailed.

However, while recent articles may include some that are much more reliable than the highly cited older papers, they have had little citation impact to date. Of the primary research papers published since 2016, the best cited [124] has an indegree of 60, and only four have an indegree of more than 40.

Two of these are small trials of intranasal oxytocin in children with autism [124,125], both reporting effects on social responsiveness but not on repetitive behaviours. As noted earlier, two similar but much larger trials, both with negative outcomes, were published in 2020 [98] and 2021 [99], too late to be cited enough to register in this analysis.

Another, by Spengler *et al.* [126], is an fMRI study of the effects of intranasal oxytocin on amygdala responses to fearful faces, expanding on the early report by Kirsch *et al.* [61]. It reports that while 12 IU of oxytocin was ineffective, 24 IU attenuated the amygdala response, while 48 IU amplified it. Responses were significant only in scans 45 min after treatment, not at either 15 or 75 min. Thus the dose window for intranasal oxytocin to attenuate the amygdala response to fear appears to be very narrow; which would seem to present a problem for any contemplated therapeutic use.

The fourth relatively well-cited recent study, by Hung *et al.* [127], reported that, in male mice, the presence of a novel juvenile increases the activity of neurons in the paraventricular nucleus that project to the ventral tegmental area—a site containing dopamine neurons that is implicated in diverse reward processes. Activating this projection (using optogenetics) was rewarding, as indicated by establishment of a conditioned place preference, and activating the projection during contact with a juvenile mouse increased the time spent in contact.

Other recent papers in rodents have also introduced methodological refinements in the study of oxytocin effects on behaviour. New approaches have enabled the activity of oxytocin neurons to be monitored in real time in conscious animals [128], and have established new experimental models for studying complex social behaviours in rodents—such as consolation behaviour in prairie voles [129], a behaviour apparently analogous to empathy in humans. These innovations are promising, opening new ways of testing hypotheses [130].

However, all new approaches will have flaws yet to be fully recognized. For example, while the optogenetic approach of Hung *et al.* would have activated the projection from the paraventricular nucleus to the ventral tegmentum, it would also have activated axon collaterals that project elsewhere—there are relatively few parvocellular oxytocin neurons in the brain, and they seem to have widespread projections. Replicating methodologically sophisticated rodent studies is also likely to be much more difficult than replicating human studies; it has become common for studies published in high-impact journals to contain diverse small elements using very different methodologies to construct a complex circumstantial narrative. The rationale for this appears to be that a claim is more convincing when supported by diverse sources of evidence. This may be true when the diverse sources of evidence all address the same issue of fact; but when the diverse sources comprise a narrative chain rather than convergent evidence, the chain itself is only as strong as its weakest link—and when papers contain multiple technological elements, not all receive equivalent scrutiny by peer reviewers.

Looking back over the last 15 years, we might note that the narrative of a prosocial role for oxytocin attracted enormous media interest at a time when scientists have been urged as never before by their funders to reach out to the public and demonstrate the social relevance of their work. For a few years, at a time when scientists were measured by their citation impact as never before, and a time when journals were desperate to increase their impact factors, papers in this field gathered an exceptional and unsustainable citation impact. And for a few years, growing despair at a lack of advances in treating autism and schizophrenia made for desperation in the hunt for new leads. It is perhaps easy to see how perfect the conditions have been for confirmation bias to take root.

## Data Availability

A note that describes the data set used for all analyses is available on https://zenodo.org/record/6615221 [131].
